# Whole-body MRI and MRA for evaluation of the prevalence of atherosclerosis in a cohort of subjectively healthy individuals

**DOI:** 10.1007/s13244-012-0180-1

**Published:** 2012-07-27

**Authors:** M. Laible, S. O. Schoenberg, S. Weckbach, M. Lettau, E. Winnik, J. Bischof, R. Franke, M. Reiser, H. Kramer

**Affiliations:** 1 Department of Clinical Radiology, University Hospital Munich, Grosshadern Campus, Munich, Germany; 2 Department of Neurology, University Hospital Heidelberg, Im Neuenheimer Feld 400, 69120 Heidelberg, Germany; 3 Department of Clinical Radiology and Nuclear Medicine, University Hospital Mannheim, Medical Faculty Mannheim, University of Heidelberg, Mannheim, Germany; 4 Department of Neuroradiology, University Hospital Freiburg, Freiburg, Germany; 5 Siemens Healthcare, Siemens AG, Erlangen, Germany; 6 Gesundheitsdienst BMW AG, BMW AG, Munich, Germany

**Keywords:** Cardiovascular disease, Epidemiology, Magnetic resonance angiography

## Abstract

**Objectives:**

To assess the prevalence of cardiovascular findings in asymptomatic individuals by means of 1.5-T whole-body magnetic resonance imaging and angiography.

**Methods:**

A cohort of 138 individuals (118 men, 20 women) with a mean age of 54 years (SD ± 7.55) was referred to whole-body MRI at 1.5-T, including contrast-enhanced whole-body MR angiography (MRA) and cardiac MRI. A total of 2,065/2,070 vessel segments (99.8%) and cardiac function were evaluated.

**Results:**

Approximately one-fourth of the participating individuals had vascular abnormalities. In 17 subjects (12.3% of all subjects) significant luminal narrowing was observed in at least one vascular segment. Luminal narrowing (mild to severe) was observed in 1 (0.7% of all subjects respectively) of the renal arteries, 7 (5.0%) of the carotid arteries, and 3 (2.2%) of the pelvic and upper leg arteries, and in 17 segments (12.3%) of arteries in the lower leg.

In cardiac function and perfusion imaging, wall motion disorders were observed in six patients (4.3%), with additional delayed enhancement and isolated delayed enhancement present in two cases. Functional parameters differed from reference values in 55 cases.

**Conclusions:**

Even in an asymptomatic cohort of middle-aged predominantly male individuals, atherosclerotic disease is not uncommon and is detectable by whole-body MRI.

***Main Messages*:**

- *In middle-aged predominantly male individuals, atherosclerotic disease is not uncommon.*
- *Even in an asymptomatic collective, approximately one fourth had vascular abnormalities.*
- *Using whole-body MR angiography (MRA), 99.8% of 2,070 vessel segments could be evaluated.*

## Introduction

The first manifestation of cardiovascular disease may occur as a fatal event such as myocardial infarction or stroke [27]. Thus, early detection and diagnosis of cardiovascular disease allow the initiation of secondary preventive measures, which seems to be most favorable in early disease stages. This has been shown to be beneficial for risk reduction related to subsequent events such as myocardial infarction and sudden cardiac death [1, 2]. The occurrence of atherosclerotic vascular disease in one vascular territory makes a manifestation in further vascular regions highly probable [3]. While complete atherosclerotic lesion reversal is usually not possible, disease progression can be controlled and decelerated.

Various diagnostic methods have been used to evaluate the presence of atherosclerosis in humans; however, only certain modalities are adequate for use in asymptomatic persons. Magnetic resonance angiography (MRA) is a compelling modality that lacks ionizing radiation, is non-invasive, and has less risk of nephrotoxicity compared with iodinated contrast agents in patients with sufficient renal function (GFR > 30ml/min). In addition, the feasibility of whole-body MRI examinations has been documented recently by several studies [4, 5, 11–16]. For tumor imaging, whole-body MRI (wb-MRI) has also been introduced and has comparable accuray to PET-CT for tumor and metastasis detection. However, wb-MRI has been predominantly used for cancer staging or follow-up examinations in subjects with a history of malignant disease and not as a primary screening tool.

Whole-body MR angiography is also feasible, and prior studies have shown that whole-body contrast-enhanced MR-angiography (wb-CE-MRA) has a high sensitivity and specificity for estimating the extent of atherosclerosis in patients with vascular disease compared to digital subtraction angiography (DSA) as the gold standard [4, 5].Therefore, the primary intent of this study was not to determine the diagnostic accuracy of wb-MRA, but to describe whole-body MRI findings in terms of atherosclerotic changes in a cohort of asymptomatic middle-aged individuals in Germany in an observational prospective study.

## Materials and methods

### Study subjects

Our study protocol was presented to and approved by the local Institutional Review Board. Subjectively asymptomatic subjects that participated in a routine health screening program and for this purpose were regularly seen by their medical practitioners were eligible for inclusion. All healthy attendants of the routine health screening program of two major German companies were asked to participate in wb-MRI/-MRA on a voluntary basis. For a precise pre-selection on the basis of self-reported patient history, we excluded all subjects with a preliminary history or symptoms of cardiovascular disease.

One hundred thirty-eight individuals, 118 males and 20 females with a mean age of 54 years (age range, 39–74 years), consented to participate, met the inclusion criteria and were thus included between April 2003 and January 2005. For the age distribution of the participants, see Tables 1 and 2. Each subject gave oral and written informed consent. The authors were not financially supported by the referring health practitioner’s companies.

**Table 1:** Data of participants (n = 138)

	N	Median age (years)	Average (years)	Standard deviation (years)
Men	118	56.0	55.6	7.3
Women	20	51.5	54.2	9.0
Total	138	56.0	55.4	7.6

**Table 2:** Graduation according to age

	Up to 49 years	50 to 59 years	60 to 69 years	70 or more years
Number of participants	33	57	44	4

As subjectively reported spontaneously by themselves, the participants' health practitioners in parallel had not documented any cardiovascular or malignant disease, but reported risk factors such as familiar predisposition, cigarette smoking, lack of exercise, etc. The basic health examination consisted of electrocardiography at rest, stress testing using an ergometer, transthoracic echocardiography, duplex ultrasound of the carotid arteries, and chest radiography. Further, an abdominal ultrasound was performed, as was blood analysis including a complete blood cell count and differential blood cell count. The findings of the whole-body MRI were classified as follows. Relevant findings were defined as those that led to a referral to a physician, and further diagnostic workup or therapy (e.g., signs of myocarditis). Non-relevant findings were defined as incidental pathological findings that did not require further monitoring (e.g., a solitary renal cyst).

Whenever data on routine or dedicated control examinations were available, MRI findings were compared on a case-by-case basis.

### MRI systems

While conducting the study, two different MRI systems were used. The first 36 consecutive subjects (group 1) were imaged using a standard clinical 1.5-T MRI system equipped with eight receiver channels (Magnetom Sonata Maestro Class, Siemens Medical Solutions, Erlangen, Germany). The following 102 consecutive subjects (group 2) were evaluated using a 1.5-T whole-body MRI system equipped with 32 receiver channels (Magnetom Avanto, Siemens Medical Solutions, Erlangen, Germany).

Since both systems were equipped with multiple receiver channels and multi-element surface coils, use of parallel imaging techniques (integrated parallel acquisition techniques, iPAT, Siemens Medical Solutions, Erlangen, Germany) was possible with both scanner types.

### Whole-body MRI protocol

To perform pre- and postcontrast imaging of the whole body, a dedicated imaging protocol had to be designed. In group 1, using a standard MR system, for imaging of the thorax, carotid arteries, and the brain, the subject was positioned head first in the magnet and the region of interest placed in the isocenter by table movement. For the second part of the MR examination, the participant was repositioned feet first in the scanner. This allowed for an examination beginning with the lower thorax down to the feet. In group 2, using a dedicated whole-body MR system, repositioning was not necessary because of the wide table movement range of up to 205 cm. All coils necessary for the exam were positioned prior to starting the examination and separately selected during the exam when needed. After acquisition of all necessary localizers, all subjects underwent non-contrast-enhanced MR imaging of the lungs first using half-Fourier RARE techniques. Second, imaging of cardiac function and perfusion was performed followed by high-resolution contrast-enhanced MR angiography as specified in Table 3 for the standard MR imager and in Table 4 for the whole-body imaging system.

**Table 3:** MRI protocol for the 1.5-T standard MR system

Examination time in min	Anatomic region	Sequence
Start	Thorax and abdomen	Half-Fourier RARE
	Heart	Function and perfusion imaging
30	Carotid arteries	MR angiography
	Brain	T1- and T2-weighted imaging
	Thorax	Volumetric interpolated breathhold examination
	Heart	Delayed contrast-enhanced imaging
60	Abdominal aorta, thigh, and calf vessels	MR angiography
90	Abdomen	Half-Fourier RARE, FLASH

**Table 4:** MRI protocol for the whole body imager

Examination time in min	Anatomic region	Sequence
Start	Thorax and abdomen	Half-Fourier RARE
	Heart	Function and perfusion imaging
30	Carotid arteries, calf vessels	MR angiography
	Brain	T1-, T2-, and diffusion-weighted imaging
	Heart	Delayed contrast-enhanced imaging
	Thorax	Volumetric interpolated breathhold examination
60	Abdominal aorta, thigh vessels	MR angiography
90	Abdomen	FLASH

### Cardiac function imaging

For assessment of cardiac function, we used a real-time cardiac imaging sequence (true fast imaging with steady-state precession, TrueFISP). In this sequence, all k-space lines are acquired within two heartbeats for imaging of a single slice of the heart. Using parallel imaging techniques, the temporal resolution could be improved to 48 ms. Miller et al. assumed a higher influence of temporal resolution to functional parameters of the left ventricle (LV) compared to the influence of spatial resolution of less than 2 mm [6]. Cardiodynamic parameters including end-diastolic volume (EDV), end-systolic volume (ESV), EF, and myocardial mass (MM) were calculated using the modified Simpson's rule (Argus Software, Siemens, Germany). All values were normalised to body surface measurements in square meters.

### Myocardial perfusion imaging

First-pass perfusion imaging of the heart at rest was performed using a saturation-recovery trueFISP sequence with a parallel imaging factor of 2. This allowed for an acquisition of four slices in a single breathhold. For myocardial perfusion imaging, a Gd-based contrast agent was used at a dose of 0.05 mmol per kilogram of body weight. Perfusion imaging at rest can only detect severe coronary artery stenosis. However, using pharmaceutically induced stress in subjects with risk factors for cardiovascular disease but without clinical symptoms was not ethically feasible.

### Cardiac delayed contrast-enhanced imaging

It is well known that after administration of Gd contrast agents, infarcted myocardium exhibits delayed hyperenhancement and can be imaged using an inversion recovery sequence. The performance of such a method when using magnitude-reconstructed images is highly sensitive to the inversion recovery time selected. The contrast agent washes in and out of the normal perfused myocardium and infarcted tissues at different rates, giving rise to the difference in tissue T1 values and leading to the observed delayed hyperenhancement [7]. We used a phase-sensitive inversion recovery sequence as this technique enables cardiac delayed contrast-enhanced (DCE) imaging without prior scout imaging. This sequence allows for the imaging of nine slices during one breath-hold and has been shown to deliver accurate identification of the area and volume of infarction with high spatial resolution [8, 24]. For best depiction of delayed enhancement, we chose a delay time of 13 to 17 min after contrast medium application, as reported in the literature.

### Contrast-enhanced MR angiography

Parallel imaging techniques with an acceleration factor of 2 were applied for acquisition of all vascular territories. This resulted in an isotropic spatial resolution below 1.5 mm^3^ for the images of each station. We decided not to include coronary artery angiography in our study protocol, as this promising technique has not yet been introduced in the routine clinical setting [9, 10]. MR angiography was performed using a standard 0.5-molar Gd contrast agent (gadopentetatedimeglumine, Magnevist, Schering, Berlin, Germany). The wb-MRA examination was divided into five stations in group 1 and four stations in group 2, respectively. Classification of arterial stenoses was as follows: mild stenosis ≤50%, moderate stenosis 50–70%, and high-grade stenosis ≥70% luminal narrowing. The first station included the aortic arch and the supra-aortic arteries. The second station imaged the abdominal aorta including the renal and iliac arteries. MRA of the lower extremities was divided into three and two steps for group 1 and 2, respectively. In subjects examined with the standard MR scanner (group 1), two different contrast injections were used to first image station 1; after repositioning of the individual, a second injection was used for stations 2 to 5. A biphasic injection protocol was used for the second injection with the first bolus consisting of 10 ml of contrast agent at a rate of 1 ml/s, followed by a bolus of 15 ml at a flow rate of 0.5 ml/s. For whole-body MRA of subjects in group 2 imaged with a dedicated whole-body MR system, two injections of contrast agent were also needed [26]. However, in this protocol, injection 1 was used to acquire stations 1 and 4 (carotid and calf arteries), while the second injection of contrast material was used for imaging of stations 2 and 3. This dedicated injection and acquisition protocol was used to minimise venous contamination while keeping the total amount of contrast agent as low as possible [24, 25].

Two radiologists with more than 6 years of experience in cardiovascular MRI evaluated the data individually. Findings were discussed and recorded according to consensus practice.

## Results

All subjects successfully completed the wb-MRA scan. No adverse events occurred. In 31% of all participants, imaging revealed findings that led to further exams or a therapeutic intervention, e.g., starting a secondary preventive medication.

### Wb-MRA vascular system

**Fig. 1:**
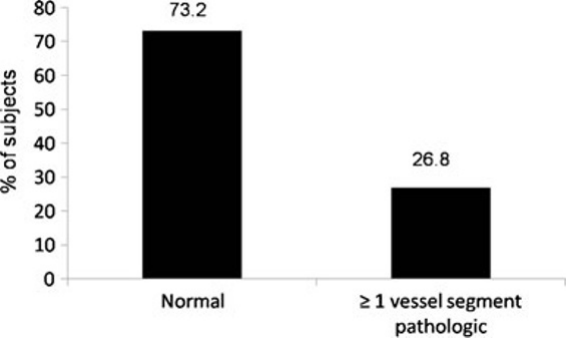
Percentage of subjects with at least one pathologically modified vessel segment

**Fig. 2:**
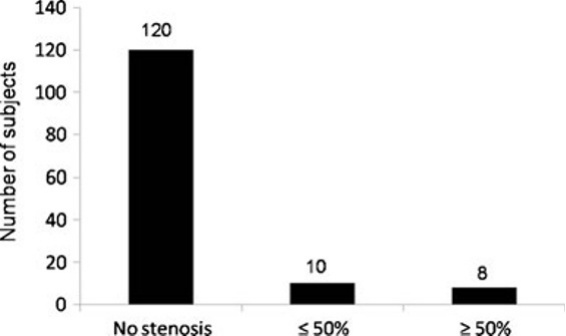
Distribution of significant luminal narrowing in the present study population: 7.2% of the individuals showed mild to moderate luminal narrowing in at least one vascular territory; another 5.8% had at least one stenosis of ≥50%; 86.9% presented without any stenoses

**Fig. 3:**
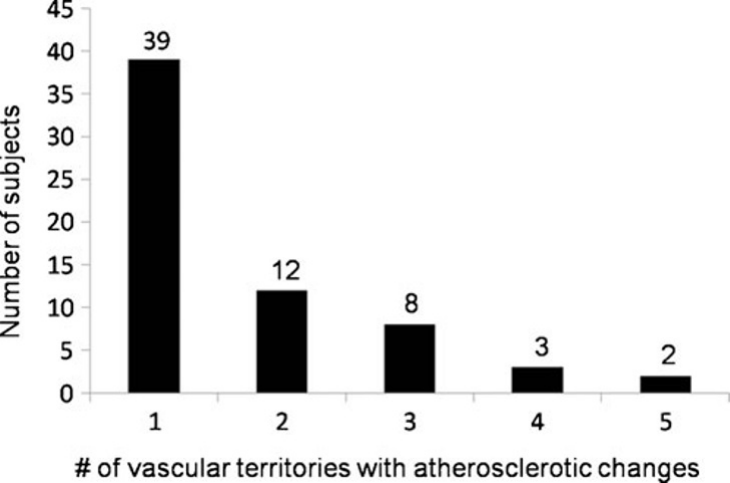
Number of vascular territories (carotid arteries, iliacal arteries, aorta, femoral arteries, tibial and fibular arteries) that presented with atherosclerotic changes (plaques, luminal narrowing, dissection, aneurysm) in one subject at a time

**Fig. 4:**
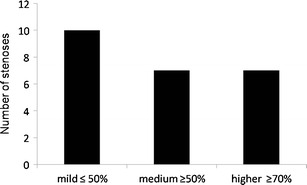
Number of stenoses of mild, medium, and higher grade found in all participants

Of all study participants, 26.8% showed pathological changes in at least one of the examined vessel segments (Fig. 1). In 18 individuals (7.2%), relevant atherosclerosis was found, i.e., at least a moderate stenosis (Fig. 2). In 39 subjects, atherosclerotic changes were present in one vascular territory. In a further 25 subjects (18.1%), stenosis was found in more than one arterial segment (Fig. 3). A maximum of five arterial vascular regions were affected (2 cases, 1.4%). Regarding carotid arteries, stenoses were observed in seven cases (4.9%). Of these stenoses, four were mild and two were moderate; in one case a higher grade stenosis of the proximal internal carotid artery was diagnosed. In the aortic region, in six cases plaques were observed, while the iliac arteries presented plaques in four cases (2.9%), but without relevant luminal narrowing. In total, there were ten mild-grade stenoses, seven medium-grade stenoses, and seven high-grade stenoses within the large arterial vessels (Fig. 4). Furthermore, we found two low-grade aortic aneurysms. Additionally, we diagnosed one infrarenal aortic dissection and a dissection of the superficial femoral artery. Considering renal arteries, one subject showed mild-grade stenosis, while another one had plaques without luminal narrowing.

**Fig. 5:**
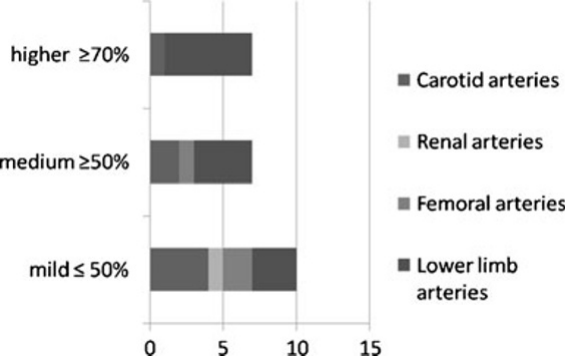
Degree of the luminal narrowing encountered. In the iliac arteries, no relevant luminal narrowing was found

The most frequent significant atherosclerotic abnormalities (>50% stenosis) were found in the lower leg, namely 12 individuals presenting with mild (2, 1.4% of all subjects respectively), moderate (3, 2.2%), or severe stenosis (7, 5.0%). The detailed occurrences of low-, medium-, and high-grade stenoses referring to the four vascular territories (carotis, renal, femoral, and lower limb arteries) are presented in Figs. 1, 2, 3, 4, and 5. The distribution of all pathological findings in the different vascular territories is summarised in Table 5.

**Table 5:** Distribution of pathological findings and non-evaluable sections in the vascular territories examined

Number of abnormal and unevaluable segments						
	CAR	AOR	REN	ILI	FEM	POP/PTA/TPT
Low grade	4		1		2	3
Medium-grade stenosis	2				1	4
Higher grade stenosis	2		1			6
Plaques	3		1	4		1
Elongation	5	4	1	2	2	
Arterio-venous shunt	1					
Aneurysm		2				
Unevaluable				1		1
Motion						
Venous overlay						
Lack of contrast						

CAR, carotid arteries; AOR, aorta; REN, renal arteries; ILI, iliacal arteries; FEM femoral arteries; POP, popliteal artery, PTA, posterior tibial artery; TPT, tibioperoneal trunk

### Cardiac MR imaging

**Fig. 6:**
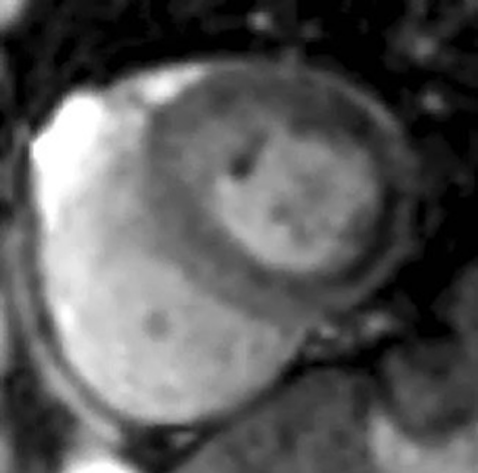
Myocardium showing a cardiac perfusion deficit detected by use of the saturation-recovery trueFISP sequence; parallel imaging factor of 2

Myocardial hypertrophy was present in four cases. Wall motion abnormalities were visually detected in six individuals (4.3%), while in one case, the examination was judged not to be diagnostic. Subendocardial delayed contrast enhancement was found in two cases (1.4%). A perfusion deficit was present in 13 cases (9.1%); for an example, see Fig. 6. Perfusion deficits related to myocardial hypertrophy were found in four cases. In five males, a regional (n = 4) or global (n = 1) myocardial dysfunction with an ejection fraction <50% was present. Nine subjects showed valve diseases (6.4%) as visually detected in the functional evaluation of the left ventricular myocardium, with two subjects displaying findings consistent with aortic valve insufficiency, two with aortic stenosis, and two with mitral valve insufficiency. In one case, signs of pericarditis were found.

### Additional findings

**Fig. 7:**
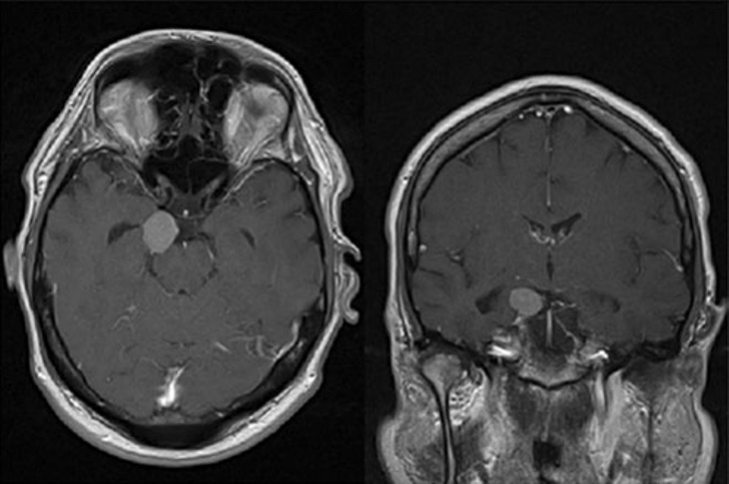
Incidental meningioma parapontine

Apart from the main target diseases, there were several incidental findings, as listed in Table 6. In the pulmonary examination, five cases of pathologically enlarged lymphatic nodules were detected. In addition, there were four inflammatory nodules, one inflammatory infiltrate, and one nodule of primarily unspecifiable origin. Concerning artheriosclerotic lesions, in three cases, cerebral microangiopathy was present. In nine further examinations (6.5%), we observed unspecific cerebral white-matter lesions, most likely due to microangiopathy. In another single case, a meningioma (Fig. 7) was detected. No malignant lesions were discovered using this cardiovascular whole-body MRI protocol.

**Table 6:** Number and characteristics of findings apart from the main target diseases

Technique	Incidental findings on MRI			Of those	
		n	%	Relevant findings (n)	Non-relevant findings (n)
Cerebral MRI	Gliosis	6	4.3		6
	White-matter lesions	9	6.5	9	
	Meningioma	1	0.7	1	
	Unspecified lesion	10	7.2		10
	Microangiopathy	3	2.2	3	0
	Atypical vessels	1	0.7		1
	Virchow-Robin spaces dilated	7	4.7		7
	Arachnoid cyst	2	1.4		2
MR angiography	Ulcer of aortic wall	1	0.7	1	
	Compression of celiactrunc	2	1.4	2	
Pulmonary MRI	Mediastinal lymph nodes enlarged	1	0.7	1	
	Hilar lymph nodes enlarged	1	0.7	1	
	Paratracheal lymph nodes enlarged	1	0.7	1	
	Axillary lymph nodes enlarged	2	1.4	2	
	Total lymph nodes enlarged	5	3.5	5	
	Pleural scars	1	0.7	1	
	Encapsulated effusion	1	0.7	1	
	Pleural layers	1	0.7	1	
	Cyst	1	0.7	1	
	Azygos lobe	1	0.7		1
	Inflammatory nodule	4	2.9	1	
	Nodule of unknown origin	1	0.7	1	
	Inflammatory infiltration	1	0.7	1	
Cardiac MRI	Aortic stenosis, low grade	2	1.4	2	
	Aortic insufficiency	2	1.4	2	
	Mitral insufficiency	1	0.7	1	
	Other valve abnormalities	4	2.9	4	
	Hypertrophy of the left ventricle	2	1.4	2	
	Pericarditis	1	0.7	1	
	Pericardic cyst	1	0.7	1	
MRI abdomen	Hepatic cysts	3	2.1	3	
	Hepatic hemangioma	4	2.8	4	
	Chronic hepatic parenchymal defect (cirrhosis, fatty liver, ascites)	2	1.4	2

## Discussion

This study showed that wb-MRI can be performed and evaluated with the use of 1.5-T clinical MR systems. The feasibility of whole-body cardiovascular MRI has been shown by several authors. General sensitivity and specificity have been determined to be between 91 to 95.3% and 90 to 95.2%, respectively [4, 5]. Until now, only a limited number of studies with larger study cohorts consisting of healthy subjects have been published [11–13]. Study collectives differ considerably; thus, caution has to be exercised when comparing results between different studies. Our study provides useful information about the prevalence of cardiovascular disease in a large, relatively homogenous collective. Therefore, it contributes data to assess the significance of wb-MRI/-MRA in broad clinical use as a diagnostic screening tool. We observed vascular pathology in more than 26% of asymptomatic subjects, and this may support the secondary preventive use of wb-MRI/-MRA in a preselected population.

Nevertheless, until now, we do not have sufficient long-term outcome data to evaluate whole-body MRI as a screening tool compared to other measures of secondary prevention. In the future, prospective studies need to be performed to answer this question. At the same time, the cost-effectiveness of the method must be investigated.

Hansen et al. reported on wb-MRA for screening for atherosclerotic disease in 307 elderly patients. In Hansen’s collective, two-thirds of the subjects had vascular abnormalities, of which one third showed stenosis of less than 50% and a third was found to have luminal narrowing greater than 50%. The remaining individuals showed no vascular abnormalities. Whereas in our cohort only 37% had vascular pathologies, one has to consider that the median age of Hansen’s cohort was 14 years older than the median age of our participants, in addition to having a different distribution of males and females. According to our results, most of relevant luminal narrowing detected by Hansen was located in the arteries of the lower legs.

Whenever we are investigating subjects with known underlying factors predisposing to atherosclerotic changes, a much higher prevalence of manifest arteriosclerosis is found. Weckbach et al. found evidence for cardiovascular disease in patients with long-term histories of diabetic disease in 49% of cases for peripheral artery disease, in 25% of cases for myocardial infarction, and in 28% of cases for cerebrovascular disease [14]. Ladd et al. analyzed patients with coronary artery disease (CAD) for additional signs of arteriosclerosis. Ten percent of the total group had symptoms of stroke once in their lifetime; 48.1% had luminal narrowing greater than 50% in at least one extracardial artery [15]. In 60 study subjects with known peripheral arterial occlusive disease, Fenchel et al. [16] found 38 subjects with myocardial dys-, hypo-, or akinesia (63.3%). This is a considerably higher percentage than the 4.3% we observed in our collective. Cardiac delayed contrast enhancement was present in 24 (40%) cases versus 2 (1.4%) cases in our study and cerebral ischemia in 18 (30%) cases vs. 1 (0.7%) case. Significant cerebral microangiopathy was present in seven (11.7%) cases vs. none in our collective.

The prevalence rates for the presence of atherosclerosis and its complications observed in our study population without a prior history of vascular disease are comparable to data known from large epidemiological studies on middle-aged subjects. Goehde et al. found relevant pathological findings in 90 out of 175 asymptomatic individuals (51.4%) who underwent wb-MRI/-MRA. In previous studies performed with ultrasound in asymptomatic populations, the prevalence of atherosclerosis in the extracranial carotid artery ranged from 5.9 to 32.8%. Carotid artery stenoses of at least 50% were found in 4 to 7.7% [17, 18].

Functional cardiac analysis in the present study found wall motion abnormalities in 3.6% of all study participants and cardiac perfusion deficits in 12 cases (8.7%), as illustrated by an example in Fig. 6. In this subgroup, the results for evidence of clinically unrecognized myocardial infarctions in healthy individuals seem relatively high compared to the results of Goehde et al., who detected visual wall motion abnormalities in 5 of 298 (1.7%) screening participants. This might be due to the combination of three different examinations in our study, including DCE, functional wall motion analysis, and perfusion analysis. In agreement with this, Breuckmann and colleagues described clinically asymptomatic delayed cardiac enhancement in 4% of a group of 102 individuals [19].

Another whole-body MRI study by Hegenscheid et al. included 200 healthy volunteers. With a median age of 48.3 years, the mean age of this cohort was lower than in our collective. In 176 subjects (88%), they detected 431 pathological findings, of which 45 (10.4%) required further clinical workup. In comparison, findings that necessitated further diagnostic workup or therapeutic steps were found in 31% of our volunteers.

In order to keep the scan time within an acceptable range (below 90 min), we did not evaluate the colon and intestine in our protocol, although this has been done by other groups such as Goehde et al. to screen for colon cancer and polyps [11]. With regard to the tumor screening part of their wb-MRI study, they found colon polyps in 12 cases (4.1%) and one incidental renal cell carcinoma. The majority of reports in the literature on wb-MRI as a screening tool for malignancy describe it as a screening tool for metastasis detection in known cancer patients, e.g., with breast or prostate cancer. MRI has been found to be equivalent regarding the detection of bone metastases compared to scintigraphy and FDG-PET [20, 21].

Whole-body MRI/MRA is a safe, reproducible technique with few side effects. In a collective of middle-aged individuals, more than one third of the participants showed previously unknown, in part therapeutically relevant findings. These findings led to an application of additional diagnostic measures or intensified control intervals in our study subjects. Therefore, in combination with the modification of cardiovascular risk factors, possibly fatal cardiovascular events might be prevented [1, 2].

Incidental findings were categorised as important/necessitating treatment or as incidental findings without the need for immediate/further treatment. If important findings were detected, those were immediately reported to the referring physician. Incidental findings without the need for further action were described in the written report.

There are various limitations to our study: first, we are subject to a certain selection bias as our study cohort consisted of employees from two big companies with the majority being male participants. However, female participants were not excluded from the evaluation since they also provide data on the presence of atherosclerotic changes in women compared to men. Study participants were relatively young with a mean age of 56 years compared to study subjects in comparable studies [11, 13, 14, 28]. The cumulative prevalence of peripheral arterial disease is estimated to be between 3 to 10% in epidemiological studies. By the age of 70 or more, the prevalence increases up to 15-20% [22, 23]. Second, a major limitation of this study is the lack of a gold standard to objectively evaluate the diagnostic accuracy of this wb-MRI/-MRA protocol. On the other hand, a preliminary study performed in our department with the same MR protocol showed not only good routine applicability, but could also confirm pathological findings on MRI/MRA by further diagnostic measures on a case-to-case basis [12]. However, the gold standard for the examination of atherosclerotic disease is still digital substraction angiography (DSA) or, in certain arterial territories, duplex ultrasound. In a collective of apparently healthy individuals, we had to avoid the use of ionizing radiation associated with DSA or CT as comparative methods. Ultrasound of arterial vessels would be a suitable alternative to conventional angiography. It is, however, limited to the visualisation of several well-accessible vascular regions such as the carotid arteries and the arteries of the upper legs. It is still time consuming and dependent on the examiner’s experience. Further, MR angiography is not the method of choice to describe vessel wall changes such as distinguishing soft and hard plaques. In well-accessible territories, duplex ultrasound may be more useful to detect such changes. Importantly, although whole-body MRA is accurate for the detection of atherosclerotic disease, no prospective studies have shown the benefit of interventions based on whole-body MRA findings. In order for wb-MRA to become widely accepted as a screening tool, outcome studies are needed.

In summary, wb-MRI and wb-MRA comprise a reliable, reproducible and easily applicable diagnostic tool for the detection of atherosclerotic disease in all major arterial territories. However, there is little probability for malignant disease in a cohort of asymptomatic subjects. Whenever there were suspicious findings in primary wb-MRI, an additional reference examination, such as a dedicated abdominal MRI, for further lesion characterisation was recommended.
